# Microcirculatory response to fluid challenge: should we prefer balanced colloids to rebalance tissue perfusion?

**DOI:** 10.1186/cc12317

**Published:** 2013-03-19

**Authors:** A Donati, E Damiani, R Domizi, C Scorcella, A Carsetti, S Tondi, R Castagnani, N Mininno, V Monaldi, P Pelaia

**Affiliations:** 1 Università Politecnica delle Marche, Ancona, Italy

## Introduction

Fluid resuscitation should improve tissue oxygenation in hypovolemia, besides restoring macrohemodynamic stability [1]. We evaluated the microvascular response to fluid challenge with different colloid solutions and its relation to macrohemodynamics.

## Methods

An observational study of patients receiving a fluid challenge (500 ml colloids in 30 minutes) according to the attending physician's decision. Before and after the infusion, sublingual microcirculation was evaluated with sidestream dark-field imaging (Microscan; Microvision Medical, Amsterdam, the Netherlands). Microvascular flow and density were assessed for small vessels [2]. The cardiac index (CI), intrathoracic blood volume index (ITBVI) and extravascular lung water index (ELWI) were measured in seven patients with PiCCO2 (Pulsion Medical System, Munich, Germany).

## Results

**Figure 1:**
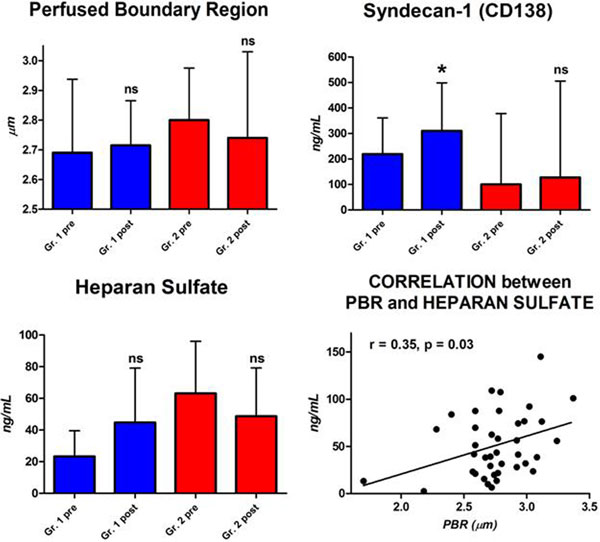
**Microvascular response to fluid challenge: effects of different colloids**.

Ten patients (two sepsis, four trauma, three intracranial bleeding, one post surgery) received either saline-based hydroxyethyl starch (HES) 130/0.4 (Amidolite^®^; B.BraunSpA; *n = *5) or balanced HES 130/0.42 (Tetraspan^®^; B.BraunSpA; *n = *5). The CI (*P *= 0.02) and ITBVI (*P *= 0.07) tended to increase, the EVLWI did not change. Microvascular flow and density improved in the whole sample. No correlation was found between macro-circulatory and micro-circulatory parameters. Balanced HES led to a greater increase in capillary density than NaCl HES (Figure 1).

## Conclusion

Balanced HES may be more efficacious than saline-based HES in recruiting the microcirculation, thereby improving tissue O_2 _delivery.
